# Diffusion of proteins in crowded solutions studied by docking-based modeling

**DOI:** 10.1063/5.0220545

**Published:** 2024-09-03

**Authors:** Amar Singh, Petras J. Kundrotas, Ilya A. Vakser

**Affiliations:** 1 Computational Biology Program, The University of Kansas, Lawrence, Kansas 66045, USA; 2 Department of Molecular Biosciences, The University of Kansas, Lawrence, Kansas 66045, USA

## Abstract

The diffusion of proteins is significantly affected by macromolecular crowding. Molecular simulations accounting for protein interactions at atomic resolution are useful for characterizing the diffusion patterns in crowded environments. We present a comprehensive analysis of protein diffusion under different crowding conditions based on our recent docking-based approach simulating an intracellular crowded environment by sampling the intermolecular energy landscape using the Markov Chain Monte Carlo protocol. The procedure was extensively benchmarked, and the results are in very good agreement with the available experimental and theoretical data. The translational and rotational diffusion rates were determined for different types of proteins under crowding conditions in a broad range of concentrations. A protein system representing most abundant protein types in the *E. coli* cytoplasm was simulated, as well as large systems of other proteins of varying sizes in heterogeneous and self-crowding solutions. Dynamics of individual proteins was analyzed as a function of concentration and different diffusion rates in homogeneous and heterogeneous crowding. Smaller proteins diffused faster in heterogeneous crowding of larger molecules, compared to their diffusion in the self-crowded solution. Larger proteins displayed the opposite behavior, diffusing faster in the self-crowded solution. The results show the predictive power of our structure-based simulation approach for long timescales of cell-size systems at atomic resolution.

## I. INTRODUCTION

Cellular environment is densely populated by various macromolecules—proteins, nucleic acids, lipids, carbohydrates, and other cellular components. The concentration of macromolecules in the cytoplasm ranges from 10% to 40% of total volume.^1^ The crowding has a significant impact on folding,^2^ stability,^3^ and kinetics of molecular interactions, including protein–protein interactions (PPIs)^4,5^ and protein–DNA/RNA interactions.^6^ It can promote protein aggregation, which may be associated with various neurodegenerative diseases.^7^ Crowding strongly impacts protein diffusion, which is essential for cellular function, including signal transduction and enzymatic activity.^8^ The crowded environment increases molecular collisions, leading to slower protein movement. The effects of crowding on protein’s diffusion are complex depending on a number of factors, including protein size and shape, concentration of the crowding molecules, and specific interactions between protein and the crowders.^9^

Experimental studies have been conducted to understand the effects of macromolecular crowding on protein diffusion using various techniques, such as Nuclear Magnetic Resonance (NMR) spectroscopy,^10^ pulsed field gradient NMR,^11^ quasielastic neutron backscattering,^12^ fluorescence correlation spectroscopy (FCS),^13,14^ single-particle tracking,^15^ and other methods.^8^ For example, FCS experiments were used to measure the translational diffusion of labeled apomyoglobin in concentrated protein solutions. The diffusion coefficient of the tracer protein in the crowded solutions was compared to its diffusion in dilute environment, and local apparent viscosities were characterized for each tracer–crowder system.^16^ The effect of macromolecular crowding on the diffusion of single molecules was investigated using size-dependent fluorescent probes. The detected diffusion depended on factors such as the viscosity range, chemical structure of the diffusing species and crowding agents, and spatiotemporal resolution of the analytical methods.^17^ In a recent study, Stadmiller *et al.*^18^ investigated protein–peptide interactions in concentrated solutions, showing the importance of physiologically relevant proteins as cosolutes for recreating crowded environments *in vitro*.

These studies revealed several important insights into the impact of crowded environments on protein diffusion. Intracellular crowding can influence the distinct diffusion behavior of different types of proteins. Globular proteins and intrinsically disordered proteins may exhibit different diffusion patterns in crowded solutions.^11^ The size of the protein affects protein diffusion in the cytoplasm of prokaryotic cells.^19^ However, these experiments do not always provide detailed mechanistic insights.^20^ The crowding can have complex effects on biochemical reactions *in vivo* compared to *in vitro*.^21^

Computational approaches to cell modeling^5,22–24^ based on hard sphere (HS) models^20^ and molecular dynamics (MD) simulations provide atomistic details of protein diffusion in dilute and concentrated solutions.^25^ Atomic resolution simulations of proteins at different concentrations have been performed to investigate protein mobility as a function of protein concentration. Using the MD simulation software GROMACS, it has been shown that high concentrations of macromolecules can influence the thermodynamics and kinetics of cellular processes, and such effects are significant in the intracellular environment.^26^ It was observed that translational diffusion slows down with an increase in the protein concentration. Rotational diffusion was found to slow down more significantly than translational diffusion at higher protein concentrations.^26^ Nawrocki *et al.*^27^ conducted all-atom MD simulations of concentrated villin headpiece solutions to analyze translational and rotational diffusion. They determined that the rotational diffusion slows down more than the translational diffusion due to the transient formation of protein clusters.

As the atomic resolution MD simulations are computationally expensive, coarse-grained simulations have been used to explore large model systems at longer timescales using different levels of structure resolution.^28^ Following the HS studies of macromolecular diffusion,^29,30^ Brownian dynamics (BD) simulations^31^ of the *E. coli* cytoplasm explored the diffusive behavior of 50 proteins from different protein families. A coarse-grained force field at amino acid resolution reproduced the diffusion slowdown in homogeneous and heterogeneous protein solutions under different crowding conditions.^32^ The results obtained from these studies provide insights into the dynamic behavior of proteins in complex cellular environments. However, these simulations are either relatively slow (timescale of ∼ns), if carried out at the all-atom representation, or significantly coarse-grained (e.g., one particle representing a protein).^23,33^

Protein docking techniques,^34–36^ which can be combined with approaches modeling large conformational changes,^37,38^ have been extensively used for structural characterization of protein complexes. Docking effectively maps the protein–protein energy landscape by determining the position and depth of the energy minima. Mapping of the intermolecular energy landscape allows speeding up simulation protocols by pre-calculating the intermolecular energy values.^39^ Our recent approach combining fast Fourier transform (FFT) accelerated systematic docking with the Monte Carlo (MC) protocol, bridging the fields of protein docking and simulation of protein interaction dynamics, enables simulation of very large protein systems with remarkable computational efficiency.^40^ The speed of calculation allows reaching seconds and longer trajectories of protein systems that approach the size of the cells, at atomic resolution. In this approach, the intermolecular energy landscape of a large system of proteins is mapped by the pairwise FFT docking^41,42^ and sampled in space and time by the Markov Chain MC protocol. The FFT-based docking algorithms have been used for decades for modeling macromolecular interactions. Recently, these methods have been employed for characterizing binding funnel^43^ and protein folding^44^ in a crowded solution. Taking advantage of its computational efficiency, in this study, we simulated various protein systems of different sizes in homogeneous and heterogeneous crowding environments. Translational and rotational protein diffusion was investigated at various concentrations and compared to diffusion in a dilute environment. This study provides a comprehensive analysis of the diffusive dynamics of proteins in concentrated homo- and hetero-protein solutions.

## II. METHODS

### A. Simulation protocol

Protein–protein interaction has been extensively studied using protein–protein docking, which generates the intermolecular energy landscape containing low-energy solutions (energy minima). These energy minima can be sampled in space and time using MC simulations. Our approach is to dramatically speed up the sampling of the intermolecular energy landscapes by skipping the low-probability (high-energy) states, focusing on the set of high-probability states corresponding to the energy minima. The intermolecular energy landscape is represented by our FFT based GRAMM docking scores corresponding to the van der Waals energies.^45^ For a system of proteins, all binary protein–protein combinations are docked at atomic resolution. The GRAMM method, widely used in the docking community, was previously optimized for docking of bound, unbound, and modeled protein structures.^46,47^ The objective of the simulation is not to seek the unique global minimum solution but to sample the vast array of transient interactions that dominate the crowded cellular environment, and the GRAMM docking energy landscape effectively provides this interaction spectrum.

The GRAMM docking was performed at an intermediate resolution (grid step 3.5 Å, repulsion 9.0, and rotation interval 10°), which accommodates small-to-medium conformational changes in proteins. Our simulation approach is based on a common protein–protein rigid-body docking approximation. The rigid-body protein–protein docking has long been recognized in the docking community as a meaningful approximation, including abundant benchmarking and blind predictions in the community-wide assessments (CAPRI).^48^ The reason for this is that most proteins undergo only small-to-medium conformational changes upon binding to other proteins, which are reasonably well accommodated by the rigid-body docking procedures with adequate tolerance to such changes. The statistics on that limited scope of conformational change have been collected for the stable complexes, corresponding to deep energy minima.^49^ On the other hand, our simulation approach focuses on transient (weak) interactions, which presumably involve even lesser extent of the conformational change, thus making the rigid-body approximation even more adequate. Still, the explicit accounting for the conformational changes would make the simulation protocol more adequate. In addition to more sophisticated force fields, that requires inclusion of the conformational transitions into the move set. Both these developments are currently on our research agenda (see Sec. IV).

The sampling of the landscape is performed using a Markov state MC protocol, according to our recently developed approach.^40^ This simulation method is designed to move a protein to an intermolecular energy minimum corresponding to binding another protein within the neighborhood. Such minima hopping paradigm is designed for crowded environments only, where proteins are next to each other, and does not hold for dilute systems. However, it allows for observation of quantitative characteristics at volume fractions as low as 0.1 to over 0.3 approximating physiological conditions.

At the initial stage of the simulation, the proteins are placed on a 500 × 500 × 500 Å^3^ grid, randomly rotated and translated within half of the grid step interval. The total number of protein copies and the step of the grid are calculated according to the preset protein volume fraction *V*. The volume fraction directly relates to the excluded volume, i.e., the space occupied by the proteins, which is useful for modeling molecular interactions and spatial distributions. In this study, we used a range of volume fraction values, from *V* = 0.10 to close to physiological *V* = 0.30.

**FIG. 1.**
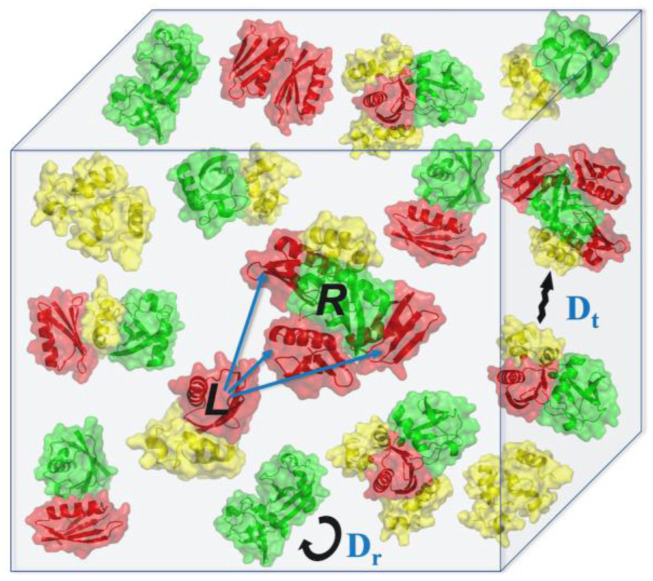
Schematic illustration of the simulation protocol. The 3-mix protein set is shown, consisting of 1pga (red), 1ubq (green), and 1vii (yellow) proteins in the simulation box. A simulation step includes a move of one protein (L, ligand) at a time to a putative docking match with another protein (R, receptor) in the vicinity of the ligand (shown by arrows). Pre-calculated docking energies, stored on 6D grids, are accessed during the MC runs. The move is accepted or rejected based on the Metropolis criterion [detailed balance condition, Eq (1)].

The position of each protein is described with the 3 × 3 rotation matrix and the translation vector relative to the origin of the coordinate system. The MC move is initiated by a random selection of a protein (ligand) considered for a move to proteins (receptors) in the proximity of the current position of the ligand. The receptor to move to is selected randomly among all the neighborhood proteins. Once the ligand and the receptor are selected, the move is selected randomly among the pre-calculated 30 000 lowest energy-docking matches for that ligand–receptor pair (Fig. 1). The simulation protocol implements periodic boundary conditions. The simulation does not involve solvent and does not consider hydrodynamics, which is subject to periodic boundary artifacts. The results did not depend on the periodic box size (see Sec. III), and thus, the finite-size corrections are not needed. For each move, the detailed balance condition was implemented. The probability *P*_*ij*_ of move from step *i* to step *j* had to be the same as *P*_*ji*_ from *j* to *i*. Accordingly, the Metropolis criterion was normalized^50^ as

$$P_{ij}=\min\left\{\;1,\exp\left[-\left(E_{j}-E_{i}\right)/T,\;\right]\times N_{i}/N_{j},\;\right\},$$ (1)

where *N*_*m*_ is the number of possible moves (receptors to move to) from state *m* with probability to be selected 1/*N*_*m*_; *E*_*m*_ is the energy of the state *m*; and *T* is the temperature of the system (a scaling factor, calibrated as *T* = 100). The simulation step was calibrated as 20 ns.^40^

Limitations of the simulation protocol, which we plan to address in future developments, include the force field restricted to van der Waals interactions, single particle MC approximation, the lack of explicit accounting for conformational flexibility, and an inherent unfavorable bias for smaller proteins in selecting neighboring proteins for MC moves at very low concentrations (at the limit of applicability of our minima hopping approach designed for crowded environment only).

The simulation protocol was extensively validated on concentrated solutions of various proteins at physiological concentrations and is consistent with experimental and modeling studies. The procedure allows simulation of extraordinarily long trajectories of cell-size protein systems at atomic resolution.^40^ For efficiency, most simulation trajectories generated for the analysis of proteins diffusion in this study were short (200 *μ*s), unless noted otherwise.

### B. Molecular systems

Simulations were performed on different sets of proteins varying from copies of a single protein (i.e., self-crowding) to a large protein system that represents most abundant protein types of *E. coli* cytoplasm. To determine the volume fraction of the system, for each protein, the protein volume was calculated by the 3V server.^51^

System 1. Three small proteins, for comparison with previous studies:^27^ ubiquitin, G protein B subunit, and villin (hereafter called the “3-mix” set).^40^
System 2. Five arbitrarily selected globular proteins of average size to represent a crowded cellular environment (the “5-mix” set).^40^
System 3. A self-crowded protein system—lysozyme (“LYZ” set), for which experimental results are available.^52^
System 4. Self-crowded systems for two proteins from the 3-mix set: a smaller protein, villin, and a larger protein, ubiquitin (“VIL” and “UBQ” sets, respectively)—well-studied globular proteins for which MD or BD results^26,27,53,54^ are available to compare the diffusion coefficients, including those of individual protein in homogeneous and heterogeneous crowding.
System 5. A large protein system that represents most abundant protein types of the *E. coli* cytoplasm.^31^ This system is composed of 37 proteins of different oligomeric state from different protein families. For system configuration and the details of proteins, see Sec. III.

Table S1 of the supplementary material contains a list of PDB IDs and the oligomeric state of the above proteins. If not specified otherwise, all protein structures were obtained from the PDB.^55^ Water, ions, and other small molecules were removed from the PDB files. In all protein systems, each protein had an equal share of copies, except for the *E. coli* set, where the share of individual proteins was determined based on the protein abundance in the *E. coli* cytoplasm.^31^ The conversion of volume fractions to g/l concentrations for each molecular system is in Table S2.

### C. Diffusion coefficients

#### 1. Translational diffusion

Translational diffusion coefficients *D*_*t*_ were obtained from the average mean square displacements (MSDs) of the protein’s geometric center. The reference position for MSD calculation was set at 2 *μ*s to allow equilibration and to avoid dependence on the initial configurations. Diffusion rates *D*_*t*_ were calculated from the slope of MSD according to the Einstein equation,

$$D_{t}=\frac{MSD\left(t\right)}{6t}$$ (2)

where *t* is the lag time.

#### 2. Rotational diffusion

Rotational diffusion coefficients *D*_*r*_ were obtained from rotational correlation time τ (global tumbling). To estimate τ, rotational autocorrelation function (ACF) was determined from the simulation trajectories with discrete time intervals Δ*t*, following the Wong and Case protocol,^56^

$$C\left(t_{i},\Delta t\right)=\left\langle P_{2}\left({\vec{v}}_{j}\left(t_{i}+\Delta t\right).{\vec{v}}_{j}\left(t_{i}\right)\right)\right\rangle ,$$ (3)

where ${\vec{v}}_{j}\left(t_{i}\right)$ is a randomly distributed unit vector (generated at time *t*_*i*_) and *P*_2_ is the second-order Legendre polynomial *P*_2_(*x*) *=* (1/2) (3*x*^2^ − 1). The unit vector was originating from the geometric center of the protein to a *C*^α^ atom. The average ACF was calculated using diffusion trajectories of 20 copies of the protein. The correlation times were then estimated by a least-square-fit of exponential function to ACF,

$$C\left(t\right)=S\;*\exp\left(\frac{-t}{\tau }\right),$$ (4)

where *S* is the order parameter and τ is the correlation time. For anisotropic molecules, the rotational behavior of the system is known to yield bi-exponentially (or triple-exponentially at highly concentrated solutions^27^) decaying correlation functions, one for fast-tumbling free proteins and the other for slower-tumbling proteins in clusters.^26^ However, we do not expect the single exponent representation to be the limiting factor in our method accuracy, considering that in our simulation only monomers are moved and that this representation is valid to the first order in anisotropy.^56^ The estimated τ was used to calculate *D*_*r*_ using the following equation:

$$D_{r}=\frac{1}{l\left(l+1\right)\tau },$$ (5)

where *l* = 2 is the order of Legendre polynomial used in ACF.

The translational and rotational diffusion rates of individual proteins were calculated at different volume fractions, from *V* = 0.10 to *V* = 0.30. Statistical errors of the diffusion coefficients were estimated from the standard deviations obtained for five replicas of each protein system. The diffusion rates as a function of volume fraction were fitted with the Cohen–Turnbull expression,^57^

$$D=D_{0}\exp\left(\frac{-\alpha V}{1-V}\right),$$ (6)

where *D*_0_ is the dilute diffusion rate and α is a constant characterizing the slowdown of the diffusion with increasing volume fraction.

Experimental and MD results are available in the literature for various, although limited, volume fractions. For some proteins, there were no data points for some volume fractions. To be consistent, we conducted simulations of different protein systems at volume fractions 0.10 to 0.30 with an interval of 0.05. Our simulation method, by design, is limited to crowded solutions (volume fraction ≥0.10). The obtained diffusion rates were fit by Eq. (6) and compared with the available experimental and MD results.

## III. RESULTS AND DISCUSSION

### A. Translational diffusion

Translational diffusion coefficients *D*_*t*_ were determined using the slope of MSD vs time (see Sec. II). Lower values of MSD slopes at higher volume fractions (Fig. S1) correspond to retarded translational motion of proteins, well established by experiment and simulation.^12,24,40^

#### 1. 3-mix and 5-mix sets

Translational diffusion coefficients of each protein type in both sets at different volume fractions are shown in Fig. S2. The results point to a pronounced slowdown of the diffusion with the increase in the protein volume fraction.^54^ Both sets have proteins of different sizes, and as reported in our previous study,^40^ the diffusion coefficients are protein size dependent (smaller proteins diffuse faster). The lowest concentration data point for the smallest protein in the 5-mix set (1cm2) was excluded (see Sec. II). To validate the absence of results dependency on the periodic box size (see Sec. II), a set of simulations was repeated at different box sizes, confirming no such dependency (Fig. S3).

#### 2. LYZ set

**FIG. 2.**
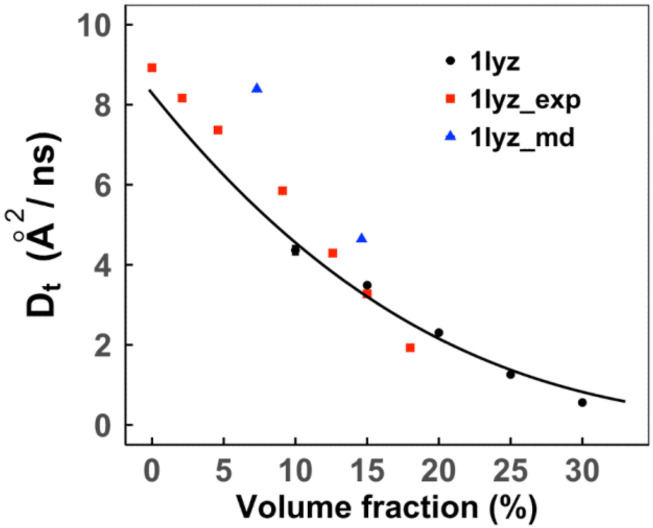
Translational diffusion coefficients of LYZ proteins in self-crowded solutions. The error bars show standard error based on five replicas simulated at each volume fraction (for some data points, the error bars are smaller than the symbol size). The data were fitted by Eq. (6) and compared with experimentally determined values^52^ and MD results.^54^

In our previous study,^40^ we validated our simulation method by determining the diffusion rates of green fluorescent protein (GFP), commonly used to analyze protein diffusion in the *E. coli* cytoplasm. The simulation of GFP with the 5-mix protein set at the physiological volume fraction was in excellent agreement with the experiment.^58^ In the current study, we extended our analysis to protein diffusion in a self-crowded environment, simulating hen egg-white lysozyme for which experimentally determined diffusion coefficient is also available.^52^ Figure 2 shows simulated diffusion coefficients at volume fractions 0.10–0.30. The experimental results were available for lower volume fractions. Thus, we extrapolated our data using Eq. (6). The results follow the expected decrease in diffusion upon an increase in the protein concentration and align well with the experimental findings.

#### 3. UBQ and VIL sets

**FIG. 3.**
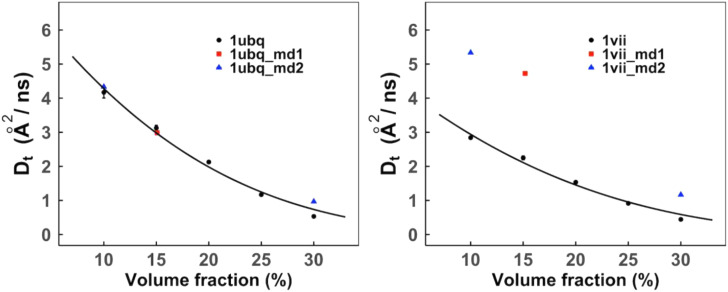
Translational diffusion coefficients of UBQ and VIL proteins in self-crowded solutions. The error bars show standard error in five replicas simulated at each volume fraction (for some data points, the error bars are smaller than the symbol size). The data points are fitted by Eq. (6). The MD results from Ref. 54 are in red, and those from Ref. 53 are in blue.

We simulated a small protein villin, and a larger protein ubiquitin from the 3-mix set in self-crowded solutions. Our choice of the VIL protein system was primarily motivated by the fact that the villin headpiece does not aggregate at moderate protein concentrations.^59^ The larger protein UBQ has a noncovalent dimer interface^60^ that indicate involvement in cluster formation.^54^ Figure 3 compares the simulated *D*_*t*_ with MD results.^26,53,54^ Most MD/BD studies report diffusion slowdown with respect to the dilute diffusion rates. Thus, there are limited data available to compare the diffusion coefficients. At physiological concentrations, a smaller protein diffuses faster in a heterogeneous crowded environment compared to its self-crowded solution. This effect is less pronounced at lower volume fractions (see Sec. III D below). Thus, both homogeneous (self-diffusion) and heterogeneous systems can be compared to each other at the lower range of concentrations (Fig. 3). The reported diffusion rates by Nawrocki *et al.*^53^ are in a heterogeneous solution (according to the authors, the rates are three times greater than in experiment and, thus, were rescaled accordingly) and closely match our estimates for UBQ at lower concentrations. The simulated diffusion rates for UBQ closely align with the self-crowded MD findings.^54^ The reported diffusion rates in Ref. 26 are higher, potentially because that simulation has only weak PPIs. For VIL, we observed the diffusion rates that are about two times slower than in Refs. 53 and 54 potentially due to overestimated VIL–VIL interactions in our simulation compared to *in vitro*, where villin headpiece does not aggregate at moderate protein concentrations. However, these rates follow the anticipated decrease with the increase of the protein concentration. More sophisticated force fields (and thus more adequate energy landscapes), application of which is currently on our research agenda, should improve the accuracy of the diffusion estimates.

#### 4. *E. coli* cytoplasm

Our simulation protocol was applied to a heterogeneous crowded solution that represents most abundant protein types of the *E. coli* cytoplasm. The composition of the macromolecules was based on an earlier computational model.^31^ The model contains 50 different types of the most abundant macromolecules in the *E. coli* cytoplasm. The BD simulations^31^ were carried out to study protein diffusion and aggregation. Since our method is currently restricted to proteins, we excluded RNA and RNA–protein complexes, primarily from large ribosomal particles. We also excluded eight large proteins with >10 000 atoms that exceeded our current procedure limitations. The resulting system comprised 37 proteins from different protein families. The 3D structures were extracted from PDB. Structures not present in PDB were modeled by SWISS-MODEL.^61^ The oligomeric state of each protein was selected as in Ref. 31. The numbers of protein copies were determined based on the chain-abundance of cytoplasmic proteins. The relative fraction was kept the same for all concentrations. The details (PDB ID, oligomeric state, etc.) of proteins in this system are in Table S1.

**FIG. 4.**
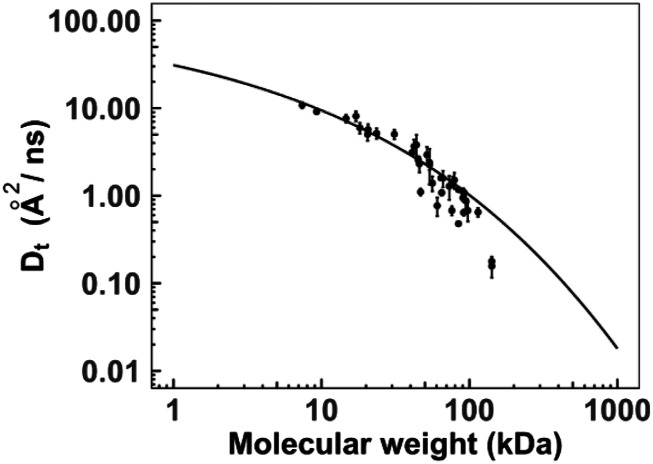
Translational diffusion coefficients in *E. coli* cytoplasm. The diffusion coefficients *D*_*t*_ were calculated for each protein by simulating a macromolecular model of *E. coli* cytoplasm (37 proteins from different *gene* families and 299 molecules in total at *V* = 0.25) as a function of protein molecular weight. The error bars show the standard error of the mean for nine replicates. The distribution was fitted by analytical expression from the experimental data (see the text).^62^

This model of *E. coli* cytoplasm was simulated at different solution concentrations, and the *D*_*t*_ for each protein was calculated. The reported macromolecular concentration in Ref. 31 was 275 g/l. Assuming the effective volume of a protein 1.0 ml/g,^1^ the total volume fraction occupied in the model is 0.27. For direct comparison of *D*_*t*_ with available experimental data on diffusion,^62^ Fig. 4 shows distribution of *D*_*t*_ according to molecular weights *Mw* of individual proteins. The computed diffusion coefficients approximate the experimentally determined trend, with slower diffusion rates for proteins of high *Mw*. The data in Fig. 4 were fitted by the following analytical expression:^62^

$$\ln\left(\frac{D_{o}}{D_{t}}\right)={\left(\frac{\xi^{2}}{R_{h}^{2}}+\frac{\xi^{2}}{r_{p}^{2}}\right)}^{\frac{-a}{2}},$$ (7)

where *r*_*p*_ is the hydrodynamic radius of the proteins, defined as $r_{p}=0.0515M_{W}^{0.392}$. *R*_*h*_ = 420 ± 90 Å and *ξ* = 5.1 ± 0.9 Å are length scales characterizing the cytoplasm, and *a* is a constant of order of one,^62^ which is the only free parameter to fit our data. The value of *a* = 0.56 for our data is in excellent agreement with *a* = 0.53 from the experimental data.^62^ The distribution of diffusion rates at other volume fractions are in Fig. S4, and the corresponding values of *a* are in Table S3.

The decrease in diffusion coefficients also scales with the size of the protein, defined by the molecular weight *Mw*, as *D*_*t*_ ∝ (*Mw*)^−*β*^ (Fig. S5). The scaling parameter *β* ranges from 0.54 to 0.80 for experimentally determined diffusion coefficients of different proteins under different crowding conditions.^63^ We obtained *β* = 0.67 and 0.85 for diffusion coefficients calculated by simulating an *E. coli* cytoplasm model system at volume fractions *V* = 0.20 and 0.25, respectively. This deviation of *β* from Stokes–Einstein diffusion theory [*D*_*t*_ ∝ (*Mw*)^−1/3^] is attributed to the fact that the cytoplasm consists of different size proteins and other crowding molecules rather than a homogenous medium of uniform viscosity.

### B. Rotational diffusion

**FIG. 5.**
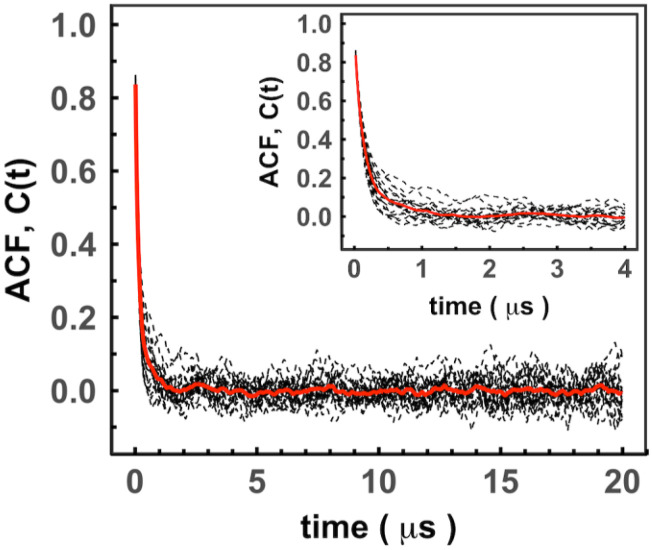
Rotational autocorrelation function. The function was calculated using scalar product of randomly distributed unit vectors with interval Δ*t* = 20 ns (see Sec. II), for 20 copies (black dashed lines) of villin in the 3-mix set at volume fraction 0.10. The inset shows variation between the correlation decays in different copies of villin and the averaged correlation function over villin copies (in red). The correlation time was estimated by a least-square-fit of exponential function [Eq (4)] to ACF.

Rotational diffusion of proteins in different systems was analyzed by evaluating rotational correlation time, τ (or rotational tumbling time) (see Sec. II). Figure 5 shows ACF with discrete time intervals of 20 ns*,* determined from the trajectories of the villin protein in the 3-mix set. The correlation times, τ, were then calculated by the least-square-fit of exponential function to ACF.

#### 1. 3-mix and 5-mix sets

Rotational diffusion coefficients *D*_*r*_ of each protein in both sets were determined using correlation time τ at different volume fractions (Fig. S6). As observed in translational diffusion, the rotational diffusion rates are lower at higher concentrations and for larger proteins. The data fit well by Eq. (6). The values of the fitting parameters *D*_0_ and α characterizing the diffusion slowdown are in Table S4. Experimental data for *D*_*r*_ are not available for the proteins in these sets. Thus, the dilute diffusion rates were compared to theoretical estimates from HydroPro^64^ at temperature 25 °C. The predicted dilute diffusion rates are in excellent match to the HydroPro predictions, with correlation coefficients *R*^2^ = 0.98 and *P* = 0.001 (Fig. S7).

#### 2. LYZ set

**FIG. 6.**
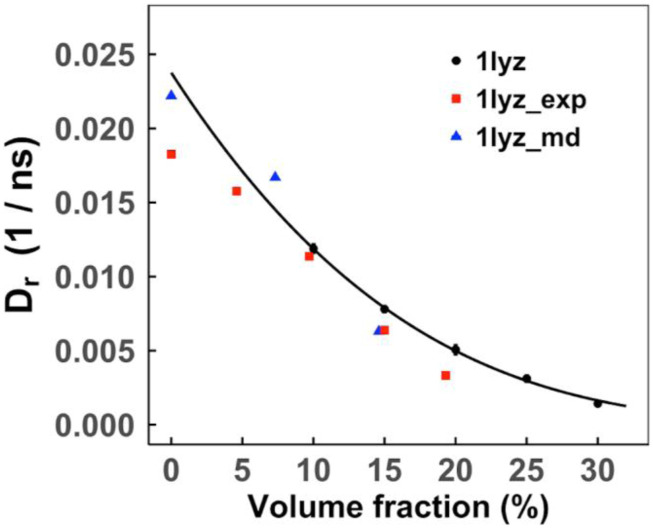
Rotational diffusion of LYZ proteins in self-crowded solutions. The error bars are standard error in five replicas simulated at each volume fraction (for some data points, the error bars are smaller than the symbol size). The data points are fitted by Eq. (6) and compared with experimental values^52^ and MD results.^54^

The simulated rotational diffusion rates were validated on the experimental data.^52^ Figure 6 compares simulated diffusion coefficients with experimentally determined values (a comparison of tumbling times is in Fig. S8; reported tumbling times^52^ were used to calculate *D*_*r*_). The rotational diffusion rates are in agreement with the experiment and the MD results^54^ with a variation of ±0.002 ns.

#### 3. UBQ and VIL sets

**FIG. 7.**
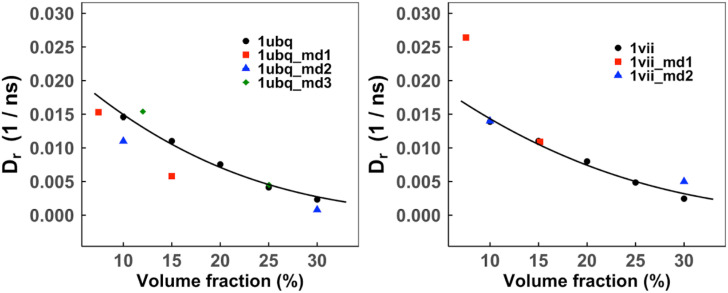
Rotational diffusion coefficients of UBQ and VIL proteins in self-crowded solutions. The error bars show standard error in five replicas simulated at each volume fraction (for some data points, the error bars are small than the symbol size). The data points are fitted by Eq. (6). MD results from Ref. 54 are in red, those from Ref. 53 are in blue, and those from Ref. 26 (UBQ only) are in green.

A comparison of our simulated *D*_*r*_ with MD results^26,53,54^ is in Fig. 7. Similar to the translational diffusion, the rotational diffusion coefficients *D*_*r*_ closely match the MD results with a variation of ±0.005 ns. While UBQ translational diffusion is faster than that of VIL at lower concentrations, the UBQ and VIL rotational diffusion rates are similar.

### C. Retardation/slowdown of diffusion

It is well known that protein diffusion in crowded solutions generally slows down with increasing macromolecular volume fraction. Molecule-specific diffusion slowdown characteristics can vary significantly between rotational and translational motions. In this section, we discuss the concentration dependent diffusion slowdown rates of individual proteins simulated in different systems. To compare the dynamics of various proteins, the diffusion rates were normalized by the infinite dilution. For each protein system, the simulated diffusion coefficients at different volume fractions were fitted by Eq. (6) with the estimated dilute diffusion rate *D*_0_. The concentration dependent normalized diffusion rate *D*^*nor*^ was computed as $D\left(V\right)/D_{0}$.

**FIG. 8.**
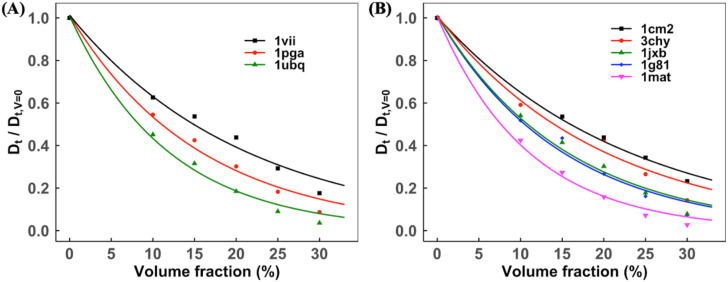
Normalized translational diffusion slowdown with increasing volume fraction. The data with the exponential fit are for the (a) 3-mix set and (b) 5-mix set. The increased concentration slows down the diffusion. At each volume fraction, the diffusion slowdown rate correlates with the size of proteins, i.e., smaller proteins have a higher normalized diffusion rate.

**FIG. 9.**
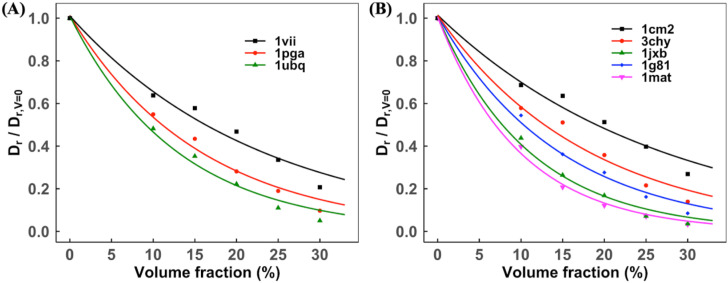
Normalized rotational diffusion slowdown with increasing volume fraction. The data with the exponential fit are for (a) 3-mix set and (b) 5-mix set. The increased concentration slows down the diffusion. At each volume fraction, the diffusion slowdown rate correlates with the size of proteins, i.e., smaller proteins have a higher normalized diffusion rate.

For each protein in 3-mix and 5-mix sets, the concentration dependent normalized translational $D_{t}^{\mathrm{nor}}$ and rotational $D_{r}^{\mathrm{nor}}$ diffusion slowdown rates are shown in Figs. 8 and 9, respectively. The results show an exponential decay of *D*^*nor*^ with increasing volume fraction, in accordance with earlier studies. $D_{t}^{\mathrm{nor}}$ correlates with the size of the protein at all volume fractions. For example, in the 3-mix set, *D*_*t*_ slows down by a factor of 0.56 and 0.81 for smaller (villin, radius of gyration *R*_*g*_ = 7.3 Å) and lager (ubiquitin, *R*_*g*_ = 11.8 Å) proteins, respectively, at *V* = 0.20 [Fig. 8(a)]. $D_{r}^{\mathrm{nor}}$ is protein-specific and appears to be related to a specific type of protein–protein interaction. For example, in the 5-mix set, $D_{t}^{\mathrm{nor}}$ is the same for similar size proteins (1jxb and 1g81, *R*_*g*_ = 15.3 and 15.0 Å, respectively) at *V* = 0.20 [Fig. 9(b)]. However, $D_{r}^{\mathrm{nor}}$ has different rates and slows down by a factor of 0.83 and 0.72 for 1jxb and 1g81, respectively. There is no literature available for proteins in these sets to compare the normalized diffusion rate *D*^*nor*^. The proteins in other self-crowded systems that we simulated are well-studied globular proteins for which MD/BD and experimental results are available for comparison. We used the reported diffusion rates in the literature. If such rates were not available, we used PlotDigitizer^65^ to extract the graphical data.

**FIG. 10.**
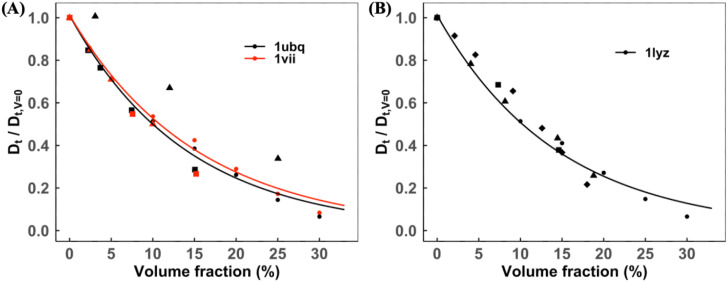
Normalized translational diffusion slowdown in self-crowded solutions. The data with the exponential fit are for (a) ubiquitin and villin and (b) lysozyme. Symbols corresponding to experimental/MD data: (a) black and red squares,^54^ black triangles,^26^ and red triangles;^27^ (b) black squares,^54^ black triangles,^68^ and black diamonds.^52^

**FIG. 11.**
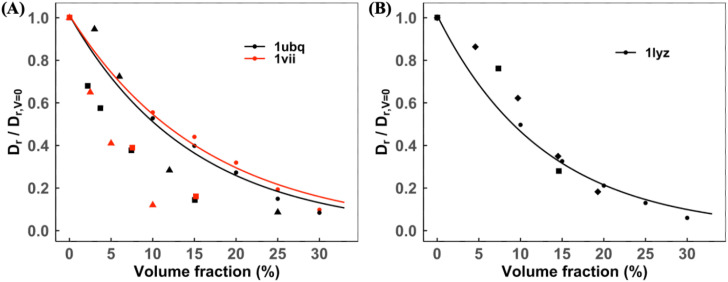
Normalized rotational diffusion slowdown in self-crowded solutions. The data with the exponential fit are for (a) ubiquitin and villin and (b) lysozyme. Symbols corresponding to experimental/MD data: (a) black and red squares,^54^ black triangles,^26^ and red triangles;^27^ (b) black squares^54^ and black diamonds.^52^

Figure 10 shows $D_{t}^{\mathrm{nor}}$ for VIL, UBQ, and LYZ proteins in self-crowded solutions. Our results are within the range of published experimental and simulation results for these proteins. As shown in Fig. 11, the normalized rotational diffusion rates $D_{r}^{\mathrm{nor}}$ from the published studies are sparse and less consistent with increasing volume concentration than $D_{t}^{\mathrm{nor}}$. Our simulation method accounts for the decrease in $D_{r}^{\mathrm{nor}}$ with increasing concentration for all proteins.

### D. Diffusion in homogeneous vs heterogeneous solutions

**FIG. 12.**
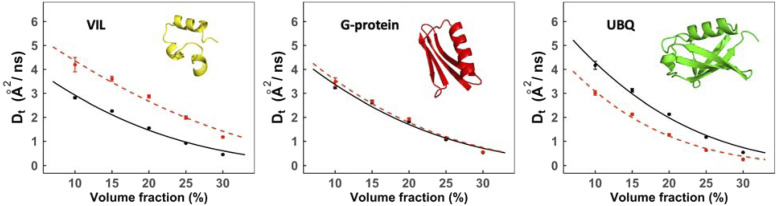
Translational diffusion in heterogeneous crowding vs. self-crowding. The diffusion coefficients of VIL, G-protein, and UBQ in the 3-mix set are in red, and those in the self-crowded solution are in black. The error bars show standard error in five replicates of each volume fraction (for some data points, the error bars are smaller than the symbol size). The data points are fitted by Eq. (6).

A recent experimental study^66^ showed that the diffusion of a smaller protein in the mixture is faster than its diffusion in a self-crowded solution, whereas the diffusion of a larger protein in the mixture is slower than its diffusion in the self-crowded solution. To investigate this, we compared the diffusion of an individual protein in the 3-mix set to its diffusing in a self-crowded solution. Our simulation results are in excellent agreement with the experiment.^66^ Figure 12 shows a comparison of the translational diffusion rates of proteins simulated in a self-crowded solution and in a heterogeneous (3-mix set) environment. The smaller protein, VIL, diffuses faster in heterogeneous crowding of larger molecules (3-mix set) compared to its diffusion in a self-crowded solution [Fig. 12(a)]. At the same time, the larger protein, ubiquitin, shows the opposite behavior, diffusing faster in the self-crowded solution [Fig. 12(c)].

We further compared the rotational diffusion rates of proteins in a self-crowded solution and in a heterogeneous (3-mix set) environment (Fig. S9). Similarly to the translational diffusion, small proteins diffuse faster and large proteins slower in a heterogeneous environment than in self-crowding. For further analysis, we calculated relative normalized diffusion slowdown rates as

$$D_{\mathrm{rel}}^{\mathrm{nor}}=\frac{{\left(D^{\mathrm{nor}}\right)}_{\mathrm{3mix}}-{\left(D^{\mathrm{nor}}\right)}_{\mathrm{self}}}{{\left(D^{\mathrm{nor}}\right)}_{\mathrm{self}}}.$$ (8)

**FIG. 13.**
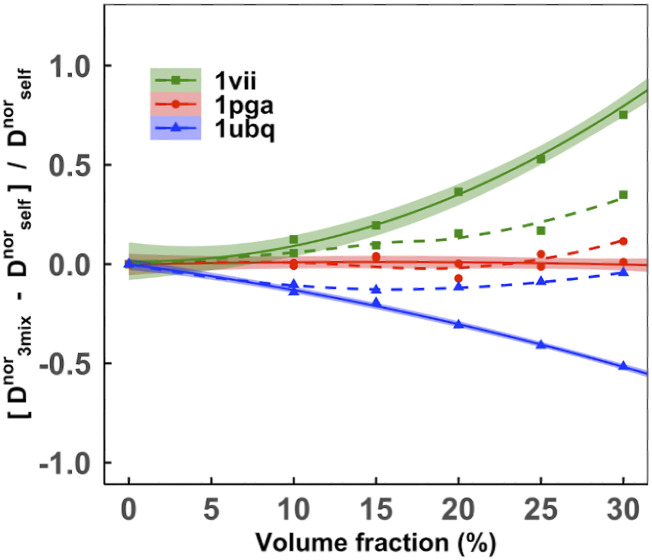
Relative normalized diffusion rates as a function of volume fraction in heterogeneous vs. self-crowded solutions. The heterogeneous crowding is in the 3-mix set. The relative translational and rotational diffusion rates are shown by solid and dashed lines, respectively. The shaded color is the confidence interval.

The relative rate as a function of volume fraction (Fig. 13) is similar to the experimental data.^66^ As the concentration increases, the disparity in translational diffusivity of the small and the large proteins becomes increasingly apparent for the translational diffusion. Notably, G-protein diffusion rates remain consistent in both crowding environments, since diffusion of particles with radius equal to an average effective radius of the macromolecular ensemble is consistent in two different solutions.^67^ The rotational diffusivity of each individual protein remains consistent except at higher concentrations. At high concentrations, UBQ shows a contrasting behavior, possibly due to the polydisperse nature of the ubiquitin, i.e., cluster formation at higher volume fractions.^54^ The average size of the UBQ cluster at higher volume fractions is larger than that of VIL (Fig. S10). UBQ has higher-order oligomeric states than VIL at high volume fractions (Fig. S11) in self-crowded solutions, which is not the case at lower volume fractions. The cluster formation increases the effective hydrodynamic radius, which hinders rotational diffusion more significantly than the translational diffusion.^27^

## IV. CONCLUSION AND FUTURE DIRECTIONS

Our recent docking-based simulation approach to atomic resolution modeling of cell-size systems was applied to the analysis of protein diffusion in crowded cell-like environments. Simulations involved large systems of proteins of various sizes. Translational and rotational diffusion coefficients were determined as a function of concentration. The results are in excellent agreement with earlier published experimental and theoretical estimates. The diffusion of proteins in heterogeneous crowding was compared to that in a self-crowded solution. Smaller proteins diffused faster in heterogeneous crowding of larger molecules, compared to their diffusion in the self-crowded solution. Larger proteins displayed the opposite behavior, diffusing faster in the self-crowded solution. The results show the predictive power of our structure-based simulation approach for long timescales of cell-size systems at atomic resolution. Future directions involve introduction of non-protein molecules, incorporation of more sophisticated force fields, multimer movement, explicit accounting for conformational flexibility, and systematic benchmarking on emerging new experimental data.

## SUPPLEMENTARY MATERIAL

See the supplementary material (Tables S1–S4 and Figs. S1–S11) for additional information on the analysis.

## Data Availability

The data that support the findings of this study are available from the corresponding authors upon reasonable request.
